# Encapsulation in Polymeric Nanoparticles Enhances the Enzymatic Stability and the Permeability of the GLP-1 Analog, Liraglutide, Across a Culture Model of Intestinal Permeability

**DOI:** 10.3390/pharmaceutics11110599

**Published:** 2019-11-12

**Authors:** Ruba Ismail, Alexandra Bocsik, Gábor Katona, Ilona Gróf, Mária A. Deli, Ildikó Csóka

**Affiliations:** 1 Institute of Pharmaceutical Technology and Regulatory Affairs, Faculty of Pharmacy, University of Szeged, H-6720 Szeged, Hungary; ismail.ruba@pharm.u-szeged.hu (R.I.); katona@pharm.u-szeged.hu (G.K.); 2 Institute of Biophysics, Biological Research Centre H-6726 Szeged, Hungary; bocsik.alexandra@brc.mta.hu (A.B.); grof.ilona@brc.mta.hu (I.G.); deli.maria@brc.mta.hu (M.A.D.); 3 Doctoral School of Biology, University of Szeged, H-6726 Szeged, Hungary; 4 Department of Cell Biology and Molecular Medicine, University of Szeged, H-6720 Szeged, Hungary

**Keywords:** liraglutide, GLP-1 analog, oral peptide delivery, enzymatic barrier, intestinal permeability, PLGA nanoparticles, Caco-2 cells

## Abstract

The potential of poly (lactic-*co*-glycolic acid) nanoparticles (PLGA NPs) to overcome the intestinal barrier that limits oral liraglutide delivery was evaluated. Liraglutide-loaded PLGA NPs were prepared by the double emulsion solvent evaporation method. In vitro release kinetics and enzymatic degradation studies were conducted, mimicking the gastrointestinal environment. The permeability of liraglutide solution, liraglutide-loaded PLGA NPs, and liraglutide in the presence of the absorption enhancer PN159 peptide was tested on the Caco-2 cell model. Liraglutide release from PLGA NPs showed a biphasic release pattern with a burst effect of less than 15%. The PLGA nanosystem protected the encapsulated liraglutide from the conditions simulating the gastric environment. The permeability of liraglutide encapsulated in PLGA NPs was 1.5-fold higher (24 × 10^−6^ cm/s) across Caco-2 cells as compared to liraglutide solution. PLGA NPs were as effective at elevating liraglutide penetration as the tight junction-opening PN159 peptide. No morphological changes were seen in the intercellular junctions of Caco-2 cells after treatment with liraglutide-PLGA NPs, confirming the lack of a paracellular component in the transport mechanism. PLGA NPs, by protecting liraglutide from enzyme degradation and enhancing its permeability across intestinal epithelium, hold great potential as carriers for oral GLP-1 analog delivery.

## 1. Introduction

The worldwide prevalence of type 2 diabetes mellitus (T2DM) has been increasing dramatically and has become a serious issue at an alarming rate. Because the incretin effect has been proven to be severely reduced or lost in relatively lean type 2 diabetic patients, incretin-based therapy, especially glucagon-like peptide 1 (GLP-1) receptor agonists, is now widely investigated for T2DM [1]. Long acting GLP-1 analogs have been developed to overcome the clinical limitations of the native GLP-1 due to its short circulating half-life [2,3].

Liraglutide (Lira; MW: 3751.2 Da) is an acylated derivative of GLP-1 that shares 97% homology to the native GLP-1, with two modifications: an Arg34Lys substitution, and a fatty acid side chain (16-carbon palmitate) attached to Lys26 via a glutamic acid linker [4,5]. Lira retains the physiological activities of GLP-1 with a considerably longer half-life (approximately 13 h) that supports once-daily dosing. Subcutaneous Lira has been EMA and FDA approved for T2DM treatment, and soon after was approved for chronic weight management [6]. As Lira is limited to administration parenterally, the development of a patient-friendly delivery should be aimed for. Herein, oral administration is the most attractive choice as this route likely mimics physiological GLP-1 secretion in addition to ensuring good patient adherence to the treatment [7,8]. Moreover, oral delivery appears to be feasible for Lira due to the relatively large safety window of GLP1 analogs compared to insulin [9]. However, the oral delivery of Lira is still challenging due to low stability along the gastrointestinal (GI) tract and poor intestinal permeability that result in low oral bioavailability [10].

The encapsulation of peptides into nanocarrier systems, especially polymeric NPs, has arisen as a very promising alternative carrier system that has greater stability in biological fluids and during storage when compared to lipid-based nanosystems [11]. Polymeric NPs can not only protect the encapsulated peptide from the harsh environment in the GI tract but also control drug release and enhance its intracellular uptake [12,13]. Poly (lactic-*co*-glycolic acid) (PLGA) nanoparticles showed potential results as nanocarriers designed for the oral delivery of insulin and exenatide [14,15,16,17].

To enhance the delivery of protein or peptide molecules across biological barriers, several strategies can be used. One of them is the opening of intercellular tight junctions [18]. Another possibility is the use of cell penetrating peptides (CPPs). CPPs comprise a family of functional carrier peptides consisting of 5–30 amino acid residues that have been reported to have great potential in enhancing the peptide drugs permeability across the intestinal epithelium. Amphipathic CPPs, such as penetratin, are among the most widely promising ones. Many recent studies proved that the non-covalent intermolecular interaction between penetratin and insulin could be clinically promising as an absorption enhancer for successful oral insulin delivery [19,20,21,22]. Our group has recently demonstrated, that a permeability enhancing 18-mer amphiphilic peptide, PN159, also known as KLAL or MAP, has a dual action: by acting on claudin transmembrane tight junction proteins it opens the paracellular route in both epithelial and blood–brain barrier models [23] and at the same time it has cell permeabilizing and penetrating properties [24].

As realizing the dream of administering antidiabetic peptides such as liraglutide orally is still an elusive goal, we reported in our previous paper the potential of implementing the quality by design methodology from the early stage of product formulation, especially when dealing with complex nanosystems such as polymeric NPs designed for peptide delivery. We have successfully optimized the formulation of Lira encapsulated in hydrophobic PLGA NPs. For the present work, the purpose was to evaluate the effectiveness of our previously developed PLGA NPs in protecting the encapsulated peptide from the harsh environment in the GI tract. As there is no available literature regarding the intestinal permeability of Lira solution, we also evaluated the permeability of Lira through the Caco-2 cell model. Moreover, we investigated the potential of the optimized PLGA NPs in enhancing the permeability of encapsulated Lira through the Caco-2 cell model and compared it with the permeability enhancer PN159 peptide.

## 2. Materials and Methods

### 2.1. Materials

Liraglutide was purchased from Xi’an Health Biochem Technology Co., Ltd. (Shaanxi, China), Poly(lactide-*co*-glycolide) (PLGA 50:50, Mw = 30,000–60,000 Da), and PVA (MOWIOL 4-98, Mw ~ 27,000 Da) were purchased from Sigma Aldrich (Munich, Germany). d-mannitol was purchased from Molar Chemicals Ltd. (Budapest, Hungary). Sodium acetate anhydrous was purchased from Scharlau Chemie S.A. (Barcelona, Spain). Ethyl acetate was from REANAL Labor (Budapest, Hungary). Pepsin from porcine gastric mucosa, powder (≥400 units/mg protein) and pancreatin from porcine pancreas (≥3× USP specifications) were purchased from Sigma Aldrich (Budapest, Hungary). All other chemicals in the study were of analytical reagent grade. 

### 2.2. Human Caco-2 Intestinal Epithelial Cell Line

The Caco-2 intestinal epithelial cell line was purchased from ATCC (cat.no. HTB-37) at passage 60. The cells were grown, as previously reported [24], in Dulbecco’s modified Eagle’s medium (Gibco, Life Technologies, Carlsbad, CA, USA) supplemented with 10% fetal bovine serum (Pan-Biotech GmbH, Aidenbach, Germany) and 50 μg/mL gentamycin in a humidified incubator with 5% CO_2_ at 37 °C. All plastic surfaces were coated with 0.05% rat tail collagen in sterile distilled water before cell seeding in culture dishes and the medium was changed every 2 days.

### 2.3. Preparation of PLGA NPs

The Lira loaded PLGA NPs were prepared by the double emulsion solvent evaporation method and then lyophilized as described previously [25]. Following the initial risk assessment-based study [26], the Plackett–Burman screening design of the experiment was applied by our research group to optimize the lyophilized Lira loaded PLGA NP formulation regarding four critical quality attributes namely: particle size, polydispersity index, encapsulation efficiency and zeta potential [25]. The optimized formula is shown in Table 1.

### 2.4. Preparation of Lira and PN159 Solutions

PN159 peptide (NH2-KLALKLALKALKAALKLA-amide) was previously synthesized and purified as reported by our research group [23,24]. To prepare Lira and PN159 solutions, liraglutide was dissolved in cell culture medium or Ringer-buffer (136 mM NaCl, 0.9 mM CaCl_2_, 0.5 mM MgCl_2_, 2.7 mM KCl, 1.5 mM KH_2_PO_4_, 10 mM NaH_2_PO_4_, pH 7.4) in a plastic vial. PN159 was weighed in a plastic vial and dissolved in Ringer buffer as well. Equal volumes of Lira and PN159 solutions were gently mixed at room temperature. The final concentrations of liraglutide and PN159 were 100 µM and 3 µM, respectively, in all experiments. 

### 2.5. In Vitro Drug Release Study

The in vitro release behavior of Lira from the prepared PLGA nanoparticles was assessed by dispersing Lira-loaded PLGA NPs (corresponding to 500 μg of Lira) in 10 mL of simulated gastric fluid without enzymes (SGFsp: 0.1 N HCl at pH 1.2). After 2 h, the NPs were centrifuged and transferred to simulated intestinal fluid without enzymes (SIFsp: phosphate buffer saline at pH 7.4).

The beaker was placed over a magnetic stirrer (100 rpm) and the temperature was kept at 37 ± 1 °C throughout the experiment. At specified time points (0, 0.5, 1, 2, 3, 4, 6 h), an aliquot of 500 μL was withdrawn from the release medium and replenished with the same volume of fresh preheated medium. Samples were centrifuged at 16,500× *g* and 4 °C for 10 min, and the Lira concentrations in the supernatant were determined by HPLC. The cumulative percentage of Lira released was calculated and then plotted versus time. All experiments were conducted in triplicate.

### 2.6. Release Kinetics Studies

To understand the in vitro release from PLGA NPs, the data were fitted into various kinetic models, which were the zero order (where the drug release rate is independent of its concentration), and first order model (where the drug release rate is concentration-dependent), and Higuchi (which describes the drug release from an insoluble matrix as a square root of the time-dependent process based on Fickian diffusion). The best-fit model was selected based on the coefficient of correlation *R*^2^. Then the release mechanism was further confirmed by fitting the release data in the Korsmeyer–Peppas equation, where the exponent (η) value was used to describe the release mechanism of the drug through the PLGA matrix; where 0.45 ≤ η corresponds to the Fickian diffusion mechanism, 0.45 < η < 0.89 corresponds to anomalous (non-Fickian) transport, η = 0.89 corresponds to case II (relaxational) transport, and η > 0.89 corresponds to super case II transport.

### 2.7. Enzymatic Degradation Study

Stability analysis in the presence of pepsin and pancreatin was conducted and compared between native Lira and Lira loaded in PLGA NPs. Five hundred micrograms of native Lira, or the amount of NPs containing an equivalent amount of Lira, were added to 2 mL of pepsin-containing simulated gastric fluid SGF (3.2 g pepsin, 2 g of sodium chloride, 7 mL HCl, mixed and diluted with water to 1 L, pH = 1.2) or pancreatin-containing simulated intestinal fluid SIF (10 g pancreatin, 6.8 g KH_2_PO_4_, mixed and adjusted with 0.2 N NaOH then diluted with water to 1 L, pH = 6.8) and incubated at 37 °C under stirring at a speed of 100 rpm. The SGF and SIF were prepared as per USP specifications (Test Solutions, United States Pharmacopeia 35, NF 30, 2012). The same methodology was followed to assess the native Lira stability in SGFsp and SIFsp. The samples (750 µL) were withdrawn at specified time intervals for 2 h, and an equal volume of ice-cold reagent was added: 0.1 M NaOH for SGF and 0.1 M HCl for SIF, to stop the enzymatic reaction. The samples were centrifuged at 16,500× *g* and 4 °C for 10 min and the supernatant was analyzed by HPLC to calculate the residual Lira. All incubations were done in triplicates. Lira recovery in the withdrawn samples was calculated using the following equation:

Lira recovery% = (Remaining Lira amount/theoretical Lira amount) × 100

### 2.8. Treatment of Caco-2 Cells

The concentration of stock solutions for cell culture experiments were 1 mM for both the therapeutic peptide liraglutide and the PN159 peptide, which was used as a reference absorption enhancer [23,24]. The working solutions were diluted in cell culture medium or Ringer-buffer depending on the experiments. The final concentration of liraglutide encapsulated in the PLGA NPs was 100 µM and was diluted directly before using. Liraglutide was examined at 100 µM, while PN159 was examined at 3 µM concentration both for cell viability and permeability. 

### 2.9. Cell Viability Measurement by Impedance

Impedance was measured at 10 kHz by an RTCA SP instrument (RTCA-SP instrument, ACEA Biosciences, San Diego, CA, USA). We have successfully tested the cellular effects of peptides and pharmaceutical excipients by impedance kinetics [23,27,28]. For background measurements, 50 μL cell culture medium was added to the wells. This was followed by seeding the cells at a density of 6 × 10^3^ cells/well to 96-well plate with gold electrodes (E-plate 96, ACEA Biosciences) coated with collagen. Cells were cultured for 5 days in a CO_2_ incubator at 37 °C and monitored every 10 min until the end of experiments. Cells were treated at the beginning of the plateau phase of growth. Lira, Lira-loaded PLGA NPs, blank PLGA NPs (without cargo), Lira and PN159 solution, and PN159 peptide were diluted in cell culture medium and the effects were followed for 24 h. Triton X-100 detergent (1 mg/mL) was used as a reference compound to obtain total cell toxicity. Cell index was defined as *R*_n_-*R*_b_ at each time point of measurement, where *R*_n_ is the cell–electrode impedance of the well when it contains cells and *R*_b_ is the background impedance of the well with the medium alone.

### 2.10. Permeability Study on the Caco-2 Model

Transepithelial electrical resistance (TEER) reflects the overall tightness of cell layers of biological barriers. TEER monitoring was performed by an EVOM volt-ohmmeter (World Precision Instruments, Sarasota, FL, USA) combined with STX-2 electrodes. The final TEER was expressed relative to the surface area of the monolayers as Ω × cm^2^ after subtraction of TEER values of cell free inserts.

Caco-2 cells were seeded onto Transwell inserts (polycarbonate membrane, 3 µm pore size, 1.12 cm^2^ surface area; Corning Life Sciences, Tewksbury, MA, USA) and cultured for three weeks [29,30]. For transport experiments, the inserts were transferred to 12-well plates containing 1.5 mL Ringer-buffer in the acceptor (lower/basal) compartments. In the donor (upper/apical) compartments, the culture medium was replaced by 0.5 mL Ringer-buffer containing treatment solutions of Lira, Lira loaded in PLGA NPs, and Lira and PN159 solution at the concentration of 100 µM for liraglutide for 1 h. Treatment solutions from both compartments were collected and the Lira level was detected by HPLC.

The apparent permeability coefficients (*P*_app_) were calculated as described previously [23]. Briefly, the cleared volume was calculated from the concentration difference of the tracer in the acceptor compartment (Δ[*C*]_A_) after 1 h and the donor compartments at 0 h ([*C*]_D_), the volume of the acceptor compartment (*V*_A_; 1.5 mL) and the surface area available for permeability (*A*; 1.1 cm^2^) using this equation:

$$P_{\mathrm{app}}\left(\mathrm{cm}/s\right)=\frac{\Delta {\left[C\right]}_{A}\times V_{A}}{A\times {\left[C\right]}_{D}\times \Delta t}$$

Recovery (mass balance) was calculated according to the equation:

$$\%\mathrm{Recovery}=\frac{C_{f}^{D}V^{D}+C_{f}^{A}V^{A}}{C_{0}^{D}V^{D}}\times 100\%$$

where $C_{0}^{D}$ and $C_{f}^{D}$ are the initial and final concentrations of the compound in the donor compartment, respectively; $C_{0}^{A}$ is the final concentration in the acceptor compartment; and $V^{D}$ and $V^{A}$ are the volumes of the solutions in the donor and acceptor compartments [29].

### 2.11. Immunohistochemistry

Aiming to investigate the morphological changes in interepithelial junctions, immunostaining for the junctional proteins, zonula occludens protein-1 (ZO-1) and β-catenin, was carried out. Cells were grown on glass coverslips (Menzel-Glaser, Braunschweig, Germany) at a density of 4 × 10^4^ cells/coverslips for 4 days and treated with Lira (100 µM), Lira loaded in PLGA NPs, Lira and PN159 solution, and PN159 peptide (3 µM) solutions for 1 h. After the treatment, the coverslips were washed with phosphate buffer (PBS) and the cells were fixed with 3% paraformaldehyde solution for 15 min at room temperature and incubated in 0.2% TX-100 solution for permeabilization. The cells were blocked with 3% bovine serum albumin in PBS and incubated with the rabbit primary antibodies, anti-ZO-1 and anti-β-catenin, overnight. Incubation with secondary Cy3-labeled anti-rabbit antibody lasted for 1 h. Hoechst dye 33342 was used to stain the cell nuclei. After mounting the samples (Fluoromount-G; Southern Biotech, Birmingham, AL, USA), the staining was visualized by a Visitron spinning disk confocal system (Visitron Systems GmbH, Puchheim, Germany).

### 2.12. Chromatographic Equipment and Conditions

Lira was analyzed by a reversed phase HPLC (Agilent 1200, San Diego, CA, USA) method that was previously developed and validated in our lab [25]. A Kinetex^®^ C18 column with dimensions of (5 μm, 150 × 4.6 mm, (Phenomenex, Torrance, CA, USA) was used as the stationary phase. The mobile phase comprised 0.02 M aqueous KH_2_PO_4_ solution (pH = 7.0, solvent A) and acetonitrile (solvent B) was pumped in a gradient mode from 80:20 (A:B, *v*/*v*) to 30:70 (A:B, *v*/*v*) in 12 min then back to 80:20 (*v*/*v*) between 12.1–15 min, at a flow rate of 1.5 mL/min. Fifty microliters of the sample was injected. The wavelength of UV detection was 214 nm. The retention time of Lira was 8.65 min.

The regression of the linearity (*R*^2^) of the Lira calibration curve was 0.996.

### 2.13. Statistical Analysis

All data presented are means ± SD. The values were compared using the analysis of variance (ANOVA) followed by the Dunnett test or the Bonferroni test, using GraphPad Prism 5.0 software (GraphPad Software Inc., San Diego, CA, USA). Changes were considered statistically significant at *p* < 0.05.

## 3. Results 

### 3.1. In Vitro Release of Lira from PLGA NPs

**Figure 1:**
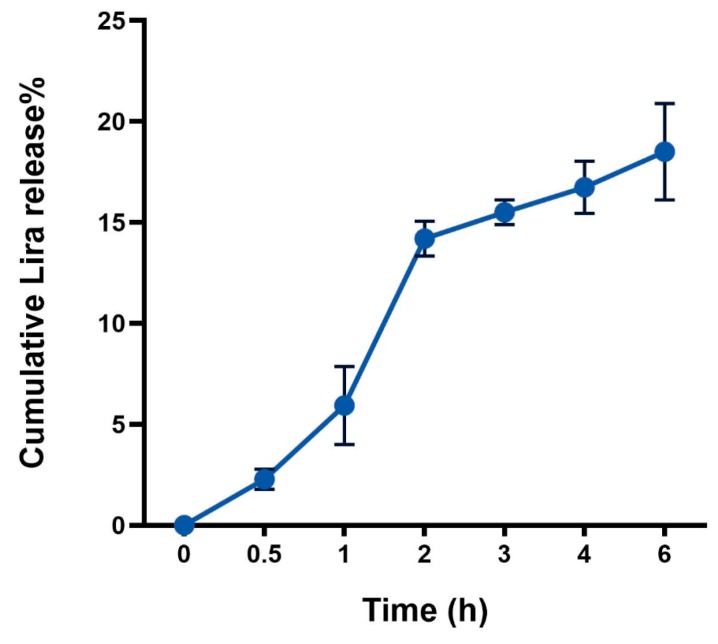
Cumulative in vitro release profile of liraglutide (Lira) from PLGA NPs.

The release behavior data presented in (Figure 1) showed a biphasic release pattern starting by a moderate initial burst release during the first 2 h in SGFsp, where 14.2 ± 0.86% of Lira was released from the NPs. This was followed by a slow release profile until 6 h in the SIFsp, where only 18.5 ± 2.39% of cumulative Lira release was reached.

### 3.2. Release Kinetics Studies

Based on the best fit with the highest correlation (*R*^2^) value, it is concluded that Lira release from PLGA NPs follows the zero-order model (*R*^2^ = 0.999) in SGFsp (pH = 1.2). When the release data is fitted into the Korsemeyer–Peppas equation (*R*^2^ = 0.999), the exponent (*n*) value is 1.316, which is consistent with the zero-order release mechanism (Table 2).

Regarding the following 4 h in SIFsp, the release mechanism follows the Higuchi model (*R*^2^ = 0.998), indicating diffusion-controlled release, and the exponent (*n*) value of the Korsmeyer–Peppas equation is 0.254, indicating that the release mechanism from PLGA NPs follows the Fickian diffusion mechanism (Table 2).

### 3.3. Enzymatic Degradation Study

**Figure 2:**
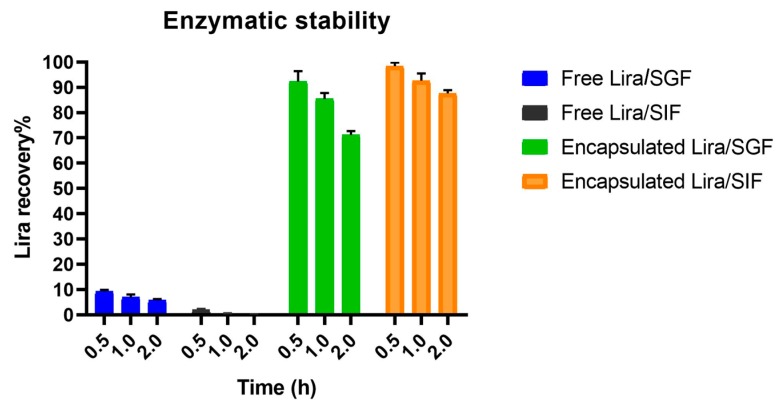
Enzymatic stability of liraglutide (Lira) encapsulated in PLGA NPs in both SGF and SIF mediums, with free Lira as a control.

It is obvious that only 1.9 ± 0.46% and 9.2 ± 0.7% of the free Lira was recovered after 30 min incubation in SGF and SIF, respectively. Lira was completely degraded after incubation for 1 h in SIF, while only 5.7 ± 0.53% Lira recovery occurred after 2 h incubation with SGF. On the other hand, the encapsulation of Lira into PLGA NPs was able to successfully protect 71.2 ± 1.49% and 87.6 ± 1.3% of Lira from degradation in the SGF and SIF at the end of the 2-h incubation, respectively (Figure 2). PLGA nanoparticles were claimed in previous research papers to be able to provide a protective and stable environment to encapsulate peptide drugs [11,31]. Encapsulation of GLP-1 into PLGA nanosystems could successfully shelter the peptide from the harsh environment of the simulated conditions of the stomach with sustained GLP-1 release [12].

### 3.4. Cell Viability Assay

**Figure 3:**
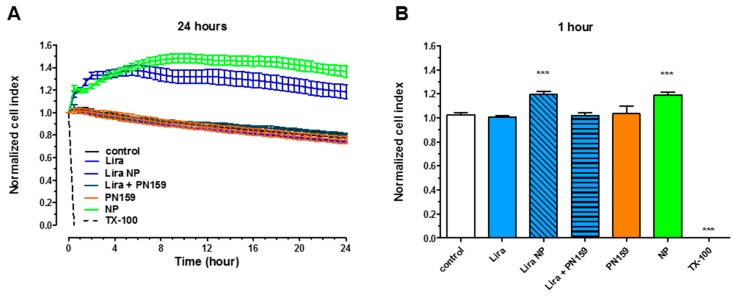
Cell viability kinetics for 24 h (**A**) and results of a 1-h treatment (**B**) of Caco-2 intestinal epithelial cells with liraglutide (Lira), NPs, liraglutide in NPs, liraglutide with PN159 peptide, and PN159 peptide measured by impedance. Values are presented as means ± SD, *n* = 6–12. Statistical analysis: Analysis of Variance (ANOVA) followed by Dunnett’s test. NPs, nanoparticles; TX-100, Triton X-100. *** *p* < 0.001 compared to control.

Treatments with Lira, Lira loaded in PLGA NPs, Lira with PN159 peptide, unloaded PLGA NPs or PN159 peptide did not change the cell index values measured by impedance, a sensitive method to detect cellular effects, indicating good cell viability (Figure 3). Figure 3A shows the kinetics of the cellular effects of the treatment solutions, while the columns in Figure 3B show the effect of treatments at the 1-h time point. When cells were lysed with the detergent Triton X-100 the impedance dropped to zero (Figure 3B).

### 3.5. Permeability Study on the Caco-2 Intestinal Barrier Model

**Figure 4:**
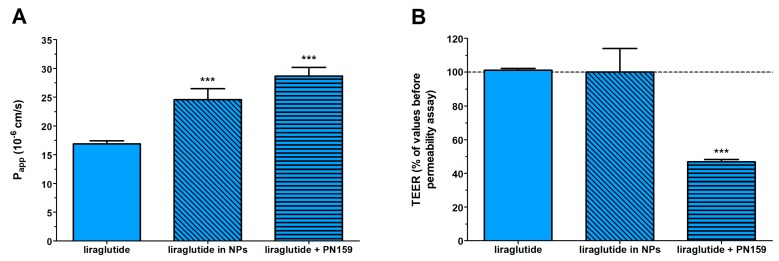
Evaluation of permeability of liraglutide (100 µM) across Caco-2 epithelial cell layers treated with different liraglutide formulations for 1 h (**A**). Changes in transepithelial electrical resistance (TEER) values of Caco-2 cell layers after 1-h treatment with different liraglutide formulations as compared to TEER values before treatment (**B**). Values are presented as means ± SD, *n* = 4. Statistical analysis: ANOVA followed by Bonferroni test, *** *p* < 0.001 compared to liraglutide group.

Caco-2 monolayers showed high TEER values (893 ± 135 Ω × cm^2^, *n* = 20) before permeability experiments indicating tight barrier properties. Because of the tight barrier the permeability was low for the marker molecule fluorescein (*P*_app_: below 0.5 × 10^−6^ cm/s) as in our previous study [29]. The free Lira at a donor concentration of 100 µM showed good penetration as the *P*_app_ was 16 × 10^−6^ cm/s (Figure 4). The permeability of Lira encapsulated in NPs, 24 × 10^−6^ cm/s, was significantly higher than that for Lira solution. An increased Lira permeability (28 × 10^−6^ cm/s) was measured in the presence of PN159 peptide, our reference absorption enhancer (Figure 4A). There was no statistical difference between the liraglutide permeability values of the Lira-NP and Lira + PN159 groups. In contrast, the only group where the TEER values dropped after the 1-h treatment was the one containing PN159 peptide (Figure 4B) indicating opening of the paracellular pathway in agreement with observations from our previous studies [23,24]. Liraglutide alone or encapsulated in PLGA NPs did not change the ionic permeability (Figure 4B), suggesting no toxic effect on differentiated Caco-2 cells in concordance with the viability data (Figure 3) and no effect on the paracellular pathway.

There was a good recovery for Lira after the permeability experiments and we found no significant differences between the recovery values of the different investigated Lira groups (Table 3). 

### 3.6. Immunohistochemistry

**Figure 5:**
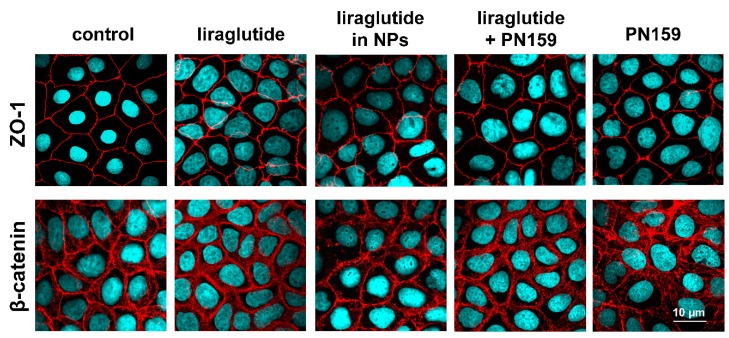
Effects of liraglutide, liraglutide in NPs, liraglutide and PN159 peptide together, and PN159 peptide on the junctional morphology of Caco-2 epithelial cells. Immunostaining for zonula occludens-1 (ZO-1), and β-catenin junction proteins after a 1-h treatment. Red color: immunostaining for junctional proteins. Blue color: staining of cell nuclei. Bar: 10 µm.

The Caco-2 intestinal epithelial cells formed confluent layers visualized by the localization of the junctional proteins ZO-1 and β-catenin. The cells were attached to each other without gaps and had similar immunostaining patterns. An intact, belt-shaped continuous localization around the cell borders for the junctional proteins was observed both in the control and the treated groups. Only the PN159 peptide treated group showed a visible change in the staining pattern of β-catenin adherens junctional protein (Figure 5).

## 4. Discussion

GLP-1 analogs represent a unique class of antidiabetic peptide drugs with potential clinical benefits over existing therapies for T2DM treatment [32]. Lira, a lipophilic long-acting GLP-1 analog, is still subcutaneously administered. Since the oral delivery of Lira can bypass the inconvenience zone of patients, a smart carrier system that can tackle the challenges hindering the oral peptide delivery has been aimed for. In a previous work, we formulated and statistically optimized the formulation and process parameters affecting the quality of Lira loaded PLGA NPs that are designed for oral delivery. Spherical shaped NPs with homogeneous distribution, 188.95 nm particle size and 51.81% encapsulation efficiency were obtained [25]. As a follow-up study, the aim of this work was to investigate the potential of the developed PLGA NPs in overcoming the main barriers limiting the oral peptide delivery, namely, the harsh environment through the GI tract and the absorption membrane barrier.

The behavior of drug release from polymeric NPs is a complex process attributed to diffusion followed by degradation and influenced by the drug physicochemical properties in addition to various formulation and process variables [33]. The Lira release from PLGA NPs showed a biphasic release pattern, which was frequently reported for polymeric NPs by previous papers [12,34]. At the burst release phase (where less than 15% of Lira was released), PLGA NPs are exposed to the gastric media and the surface of the NPs is hydrated. Then, non-capsulated Lira, or Lira which exists close to the surface having weak interactions with it is easily accessible by hydration and is released in the media. Potentially, less than 15% of Lira was released from NPs in the gastric media following a zero-order model. At the second phase of slow Lira release, the degradation of the polymer matrix took place, leading to diffusion of the encapsulated Lira, and the release mechanism followed the Higuchi model, which further confirms the diffusion-controlled release. These results prove that the PLGA nanosystem is able to hinder the release of encapsulated peptides in gastric simulating conditions without enzymes (SGFsp), and later sustain the peptide release in intestinal simulating conditions without enzymes (SIFsp).

The enzymatic stability results were compared between the free Lira and Lira encapsulated in NPs. There was no Lira detected after the 2-h incubation of free Lira with SGF or SIF, which is due to the presence of amino acids, especially the aromatic ones, in the Lira structure, which makes it vulnerable to pepsin–pancreatin digestion [35]. The results revealed that PLGA NPs were effective in protecting 71% and 87% of Lira from pepsin and pancreatin digestion after a 2-h incubation with SGF and SIF, respectively. These findings confirm that PLGA NPs can provide a physical barrier between the encapsulated Lira and the harsh environment in the GI tract, and thereby are promising for obtaining higher oral peptide bioavailability [12,36].

There was no decrease in cell impedance kinetics regarding the five treatment groups; Lira, Lira loaded in PLGA NPs, Lira and PN159 peptide in solution, unloaded PLGA NPs or PN159 peptide, during the 24-h long treatment. These findings proved the biocompatibility of PLGA NPs and showed that the composition of the nanosystem did not contribute to toxicity in Caco-2 cells. This is in accordance with previous reports where PLGA nanosystems larger than 100 nm did not trigger any toxic effects at different concentrations [37,38,39]. Regarding PN159 peptide, the absence of cytotoxic effects at the concentration of 3 µM is also in accordance with our previous report [24,40].

To further evaluate the potential of the prepared nanosystem, permeability studies on the Caco-2 cell model were conducted. Lira showed a good apparent permeability through the cell model compared to what was reported for the native GLP-1 or exenatide, which could be due to the 16-carbon fatty acid chain that is attached to lysine at position 26 via a glutamic acid spacer [9]. This acylation leads to higher Lira hydrophobicity [41], which can enhance the intracellular permeability of this GLP-1 analog when compared to the native GLP-1 or exenatide [42]. The potential of the optimized polymeric NPs was also investigated to further enhance Lira permeability. Lira encapsulated in NPs showed 1.5-fold higher apparent permeability as compared to Lira alone. Because PLGA NPs are more lipophilic compared to the free peptide drug, their transport across the lipid membrane of Caco-2 cells is better. Furthermore, these optimized polymeric NPs showed a smaller size than 200 nm, and it was previously reported that NPs within the 100–200 nm size range showed the best properties for cellular uptake, while smaller-sized (50 nm) or larger-sized (≥500 nm) particles resulted in reduced uptake [43]. Moreover, the prepared NPs were spherical with a smooth surface, as previously confirmed by scanning electron microscopy [25], which is better for cellular uptake when compared to needle-shaped ones. The ability of PLGA NPs to enhance the in vitro permeability of the hydrophilic drug alendronate [44], salmon calcitonin [45], bovine serum albumin [46], and insulin [15,17] through the Caco-2 cell model has been reported in the literature. In the presence of PN159 peptide, our reference absorption enhancer, Lira permeability also increased. This finding is consistent with our results, and results from the literature, on the reversible tight junction opening by the PN159 peptide in biological barrier models, which effectively improves the permeability of different drugs and hydrophilic marker molecules through the paracellular pathway [23,24,40]. In addition, it was reported that this amphipathic CPP can strongly bind to and interact with biological membranes due to electrostatic and hydrophobic interactions [47,48], and we also confirmed the cell permeabilizing and penetration effects of PN159 on Caco-2 cells [23].

The immunostaining for the junctional proteins, ZO-1 and β-catenin, showed that Lira-PLGA NPs did not change the morphology of interepithelial junctions. The PN159 peptide treated group showed a change in the staining pattern of the β-catenin adherens junctional protein, which is also in accordance with our previous results [23,24]. We suppose that an increase in both the paracellular transport and the membrane permeability in Caco-2 cells by PN159 can contribute to the enhanced Lira permeability.

## 5. Conclusions

Being a relatively new GLP-1 analog, there are no reported studies for Caco-2 permeability of liraglutide alone or encapsulated into a carrier system. In this study we found that the developed PLGA nanosystem could efficiently protect the encapsulated liraglutide from the conditions simulating the harsh environment in the GI tract. This polymeric system seems to be promising as it can also enhance the permeability 1.5-fold compared to free liraglutide solution. These findings reveal that encapsulation in a polymeric nanosystem holds promise for oral GLP-1 analog delivery. 

## Figures and Tables

**Table 1:** Optimized Lira loaded PLGA NPs; where PVA is the polyvinyl alcohol-stabilizer, and W2/O is the outer aqueous phase to organic phase ratio.

Formulation Parameters	
PLGA amount	60 mg
Lira amount	5 mg
2nd sonication time	0.5 min
PVA amount	1.48%
Lyoprotectant type	Mannitol
Lyoprotectant amount	5%
W2/O ratio	5

**Table 2:** Release kinetics data of Lira from PLGA NPs, in SGF and SIF (without enzymes).

Kinetic Model	SGF			SIF		
	*N*	*k*	*R* ^2^	*n*	*k*	*R* ^2^
Zero order	7.9857	1.84	0.9991	0.9836	12.658	0.9925
First order	0.038	2.01	0.998	0.0052	1.9418	0.9934
Higuchi	17.049	10.262	0.9854	4.1563	8.3559	0.9983
Korsmeyer-Peppas	1.3162	0.762	0.9993	0.2456	1.0696	0.9996

**Table 3:** Recovery (mass balance) calculation after liraglutide permeability on Caco-2 cells.

Liraglutide	Recovery (%) Mean ± SD
Liraglutide	80.9 ± 1.6
Liraglutide in NPs	75.3 ± 2.3
Liraglutide + PN159	81.3 ± 6.9
